# Correction: Mitochondrial oxodicarboxylate carrier deficiency is associated with mitochondrial DNA depletion and spinal muscular atrophy–like disease

**DOI:** 10.1038/s41436-019-0506-1

**Published:** 2019-04-26

**Authors:** V. Boczonadi, M. S. King, A. C. Smith, M. Olahova, B. Bansagi, A. Roos, F. Eyassu, C. Borchers, V. Ramesh, H. Lochmüller, T. Polvikoski, R. G. Whittaker, A. Pyle, H. Griffin, R. W. Taylor, P. F. Chinnery, A. J. Robinson, E. R. S. Kunji, R. Horvath

**Affiliations:** 1 Wellcome Centre for Mitochondrial Research, Institute of Genetic Medicine, Newcastle University, Newcastle upon Tyne, UK; 2 Medical Research Council Mitochondrial Biology Unit, University of Cambridge, Cambridge Biomedical Campus, Cambridge, UK; 3 Wellcome Centre for Mitochondrial Research, Institute of Neuroscience, Newcastle University, Newcastle upon Tyne, UK; 4 Leibniz Institute of Analytic Sciences (ISAS), Dortmund, Germany; 5 UVic-Genome BC Proteomics Centre, Vancouver, BC Canada; 6 Department of Paediatric Neurology, Royal Victoria Infirmary, Newcastle upon Tyne Foundation Hospitals NHS Trust, Newcastle upon Tyne, UK; 7 Institute for Ageing and Health, Newcastle University, Newcastle upon Tyne, UK; 8 Institute of Neuroscience, Newcastle University, Newcastle upon Tyne, UK; 9 Department of Clinical Neurosciences, University of Cambridge, Cambridge, UK; 10 Medical Research Council Mitochondrial Biology Unit, University of Cambridge, Cambridge Biomedical Campus, Cambridge, UK; 11 Wellcome Centre for Mitochondrial Research, Institute of Genetic Medicine, Newcastle University, Newcastle upon Tyne, UK

Correction to: *Genetics in Medicine* **20**:1224–1235; 10.1038/gim.2017.251; Article published online 08 March 2018

This Article was originally published under Nature Research’s License to Publish, but has now been made available under a [CC BY 4.0] license. The PDF and HTML versions of the Article have been modified accordingly.

